# Risk factors for traumatic and non-traumatic lower limb pain among preadolescents: a population-based study of Finnish schoolchildren

**DOI:** 10.1186/1471-2474-7-3

**Published:** 2006-01-18

**Authors:** Ashraf El-Metwally, Jouko J Salminen, Anssi Auvinen, Hannu Kautiainen, Marja Mikkelsson

**Affiliations:** 1 Tampere School of Public Health, FIN-33014 University of Tampere, Tampere, Finland; 2 Department of Physical and Rehabilitation Medicine, University Hospital of Turku, P.O box 52, 20520 Turku, Finland; 3 Tampere School of Public Health, and The Pediatric Research Center, FIN-33014 University of Tampere, Tampere, Finland; 4 The Rheumatism Foundation Hospital, Pikijärventie 1, 18120 Heinola, Finland; 5 Department of Physical and Rehabilitation Medicine, The Rheumatism Foundation Hospital, Pikijärventie 1, 18120 Heinola, Finland

## Abstract

**Background:**

The child's lower limb is the most commonly reported musculoskeletal location with pain and also the most commonly injured site in sports. Some potential risk factors have been studied, but the results are inconsistent. We hypothesized that distinction of traumatic from non-traumatic pain would provide a clearer picture of these factors. The aim of this study is to assess factors associated with lower extremity pain and its impact on preadolescents in a population-based cohort.

**Methods:**

A structured pain questionnaire was completed by 1756 schoolchildren of third and fifth grades to assess musculoskeletal pain, psychosomatic symptoms, subjective disabilities, school absence and frequency of exercise. In addition, hypermobility and physical fitness were measured.

**Results:**

The knee was the most common site of pain followed by the ankle-foot and thigh. Of the children who reported pain in their lower extremity, approximately 70% reported at least one disability and 19 % reported school absence attributed to their pain during the previous three-month period. Children with traumatic pain had a higher subjective disability index than those with non-traumatic pain (P = 0.02). Age less than 11 years, headache, abdominal pain, depressive feelings, day tiredness, and vigorous exercise were more common in children with lower limb pain than those free of it. In the stratified analysis, younger age was related to both traumatic and non-traumatic pain groups. Vigorous exercise was positively associated with traumatic pain, while subjects with non-traumatic pain had more frequent psychosomatic symptoms.

**Conclusion:**

Risk factors and consequences of traumatic and non-traumatic lower limb pain are not similar. Traumatic lower limb pain is associated with practicing vigorous exercise and high level of physical fitness, while non-traumatic pain is more correlated with psychosomatic symptoms. These differences might be one of the reasons for the discrepancy of previous research conclusions. The two conditions need to be treated as different disorders in future studies.

## Background

Previous studies about pediatric pain have consistently found that the lower limb is the most commonly reported musculoskeletal location with pain and has been ranked among the leading general pain symptoms in childhood and adolescence [1-3]. The lower limb is also the most commonly injured site in sports [4] and accounts for a substantial percentage of pediatric rheumatology and orthopedic consultations [5].

Two studies have reported consequences and disabilities related to lower limb pain in schoolchildren, both limited to non-traumatic knee pain. Fairbank et al. found that 18% of children aged 13–17 with knee pain had to stop playing during the past year because of their symptoms. In addition, 22% of girls and 40% of boys needed to visit a doctor [6]. In a Finnish study [7], 45% of schoolchildren aged 9–15 reported moderate to severe disability attributed to lower limb pain during past three months. Despite these high figures, less attention has been given to determinants of lower limb pain in children compared to other pediatric pain symptoms. This might be due to a belief that lower extremity pain in children is a transient, self-limiting condition [8]. Further, the substantial burden of other pain symptoms in adults [e.g. lower back pain [9] and headache [10]] has directed research interests in understanding the origin of these symptoms in childhood. However, lower limb pain may limit children's activities and lead to adoption of an inactive lifestyle persisting to adulthood [11]. Hence, more studies are needed to provide further insight into factors that influence the occurrence of this condition in schoolchildren.

Some potential determinants of lower limb pain have been studied in children and adolescents, but the results are inconsistent. A positive relationship has been reported between frequency of physical exercise and lower limb pain in most studies [6,7], but no correlation was observed in a recent follow-up study [11]. Similarly, conflicting results have been reported for the predictive role of physical fitness [12,13] hypermobility/hyperflexibility [11,14,15] and psychosocial factors [16,17]. We hypothesized that distinction of traumatic from non-traumatic pain would provide a clearer picture of the risk factors for lower limb pain.

The aims of this study were (a) to determine the prevalence of pain symptoms in different lower limb parts in a population-based sample of preadolescents, (b) to examine the associations of traumatic and non-traumatic lower limb pain with age, sex, hypermobility, frequency of exercise, physical fitness, and psychosomatic factors, (c) to assess school absence and subjective disabilities attributed to traumatic and non-traumatic lower limb pain.

## Methods

The initial study took place in Lahti, a town of 94 827 (1995) inhabitants in southern Finland. All the 21 primary schools in town were asked to participate in the survey. Two schools refused and 19 took part in the study. The Steiner school, the hospital school, and the schools for hearing and physically disabled and the mentally handicapped were excluded because some of the methods used in this study were not suitable for their pupils. All pupils from the third and fifth grades participated in the survey except those who were not at school on the day of the study [18,19].

**Figure 1:**
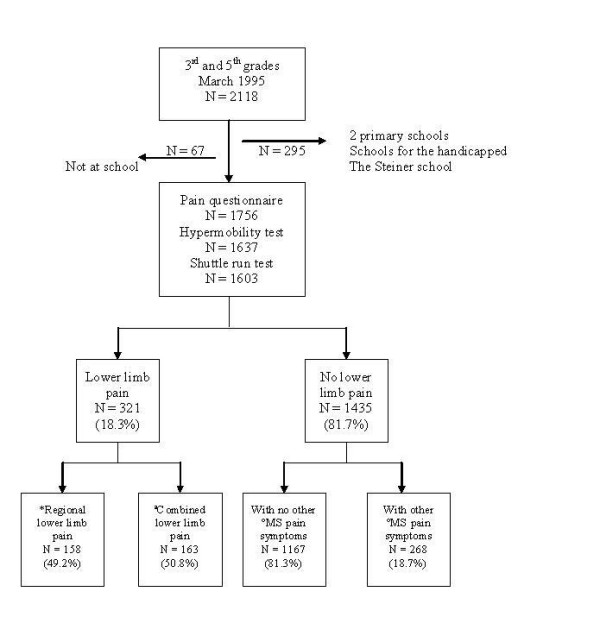
**Flow chart of the study**. *Children who reported lower limb pain as the only musculoskeletal pain symptom. ^a^Children who reported lower limb pain combined with other musculoskeletal pain symptoms ^°^Musculoskeletal pain.

The study was approved by the Ethics Committee of the Health Care Center of the City of Lahti. The parents were informed about the study by letters in advance. Both the children and their parents had the possibility to refuse enrollment in the survey. The final sample consisted of 1756 preadolescents, of whom 867 were third-grade (mean age 9.8 [SD = 0.4] years) and 889 were fifth-grade (mean age 11.8 [SD = 0.4] years) school children. This population represents 82.9% of all schoolchildren in these grades attending normal or special schools in Lahti (Figure 1). In March 1995, these children completed a pain questionnaire, during a class lesson, to assess lower limb pain during the preceding 3 months and were tested for hypermobility and cardio-respiratory fitness.

### Instruments

#### 1- Pain questionnaire

A structured pain questionnaire was designed to assess lower limb pain, as well as other musculoskeletal pain symptoms (neck, upper limb, chest, upper back, lower back, buttock) during the previous three months. Musculoskeletal pain symptoms were classified according to pain frequency (seldom or never, once a month, once a week, more than once a week, almost daily). The 5-level frequency classification was adopted from the questionnaire used in the nationwide survey on health and health-related behaviors in schoolchildren by the WHO [20]. The body area concerned was indicated on a figure placed next to the question to help the child recognize the named area. Furthermore, the children were asked to mark the exact site of pain in the lower extremity (foot, ankle, leg, knee, thigh and hip). If the child had pain due to a direct trauma (e.g. injured when exercising, fallen down, stumbled), they were asked to indicate the traumatized area on the pain drawing with a different color [18,19]. The lower extremity area shown in the picture included the area from the level of the hip joint to the toes anteriorly and the area from the gluteal folds to the toes posteriorly.

Psychosomatic symptoms (headache, abdominal pain, depressive mood, day tiredness, difficulties in falling asleep, waking up during nights) were asked about with the same frequency categorization as for musculoskeletal pain. Presence of each symptom was defined as occurrence at least once a week. Disability due to pain was assessed by the following questions (A) *do you have difficulties in falling asleep because of your pain or does your pain disturb your sleep *(B) *do you have difficulties while sitting during lessons *(C) *do you feel pain if you walk more than one kilometer *(D) *do you feel pain during physical exercise class *(E) *does your pain interfere with your hobbies*. A subjective disability index (1 point for each, maximum 5) was calculated from answers to these questions. Absence from school during the preceding 3 months due to pain or aches was asked about through the questionnaire. In addition, the children were asked about the frequency with which they undertook vigorous exercise, for at least half an hour, during the preceding 3 months and were categorized into 3 groups (0–2, 3–4, and 5–7 times a week).

During the questionnaire development, two versions of the questionnaire were tested and the test-retest reliability of the final version in detecting those who have pains at least once a week was excellent (kappa 0.9) [18]. The concurrent validity of the pain questionnaire was examined by comparing it with interviews of 31 third- and 25 fifth-grade children on the same day of the survey. The agreement between pain questionnaire and interview with similar pain questions was 86% (95% confidence interval [95% CI] 74 to 94%) and kappa was 0.67 [19].

#### 2- Hypermobility test

Hypermobility test was conducted using Beighton's method (score 0 to 9) [21]. A nurse, specially trained for the tests, examined the children during school lessons. No stretching was allowed before the test. Intra-and inter-observer reliability were evaluated earlier with kappa coefficients of 0.75 and 0.78 respectively [18]. Out of 1756 children included in the analysis, 1637 (93.2%) were examined for hypermobility. School absence on the examination date was the reason for non-participation. We used Beighton score of six as the cutoff point for hypermobility on the basis of the distribution of the results. Children were also categorized into two groups (with and without regional knee hypermobility) in part of the analysis.

#### 3- Shuttle run test

The 20-meter shuttle run test [22] is an indoor test of maximal performance. It provides a valid and reliable index of cardio-respiratory endurance or maximal oxygen uptake (VO_2 _max) [23]. It was carried out for all the study subjects excluding children with acute musculoskeletal injury, acute respiratory infection, or other diseases inhibiting maximal physical strain. Children from schools that did not have room for the 20-meter distance required for the test, were also excluded. Out of 1756 children, who were included in the analysis, 1204 (68.6%) underwent the test. Based on the distribution of the results (median 51.1 ml/kg/min), children were categorized into 3 groups according to their VO_2 _max measurements: low (below the 25^th ^percentile), average (from the 25^th ^till the 75^th ^percentiles) and high (above the 75^th ^percentile).

### Case definition of lower limb pain

Those who reported pain in their lower extremity, occurring at least once a week during past three months fulfilled our case definition of lower limb pain. Furthermore, children with lower limb pain were divided into those with traumatic and non-traumatic pain according to whether or not they have reported a direct trauma. The same case definition was used for other musculoskeletal pain symptoms. Descriptive information about these symptoms has been reported earlier [19].

### Statistical methods

Assessment of subjective disabilities and school absence due to lower limb pain was limited to those who had the lower limb as the only musculoskeletal pain area (regional lower limb group) to eliminate possible effect of other musculoskeletal pain symptoms. Differences between the two lower limb pain groups (traumatic and non-traumatic) related to their reported subjective pain disability index and school absence were assessed by t-test and Fisher's exact tests, respectively.

Logistic regression analysis was used to identify factors associated with lower limb pain in preadolescents. The logistic models included the following independent variables: age (below/above 11 years), psychosomatic symptoms (present/absent), frequency of exercise (0–2/3–4/5–7 times a week), Beighton score of hypermobility (below/above six), and VO_2 _max (low/normal/high). For analytic purposes, a new variable was created categorizing all children included in our survey into 2 groups according to the occurrence of musculoskeletal pain in locations other than the lower limb. The logistic models were first fitted with each of the independent variables separately, together with the "Other musculoskeletal pain symptoms" variable. A multivariate analysis was then conducted using a stepwise backward elimination method. All independent variables were initially included in the regression equation together with the "Other musculoskeletal pain symptoms" variable. Elimination of variables, at each step, was based on the likelihood ratio test at 10% level of significance. Interactions between variables were tested all through the analyses. All statistical analyses were performed using SPSS (for Windows), version 10.0.

## Results

### Prevalence of lower limb pain according to location

Out of 216 children who had non-traumatic lower limb pain, 95% (N = 205) reported that their pain was diffuse and not limited to one specific lower limb site. On the other hand, only 21% (N = 22) of the traumatic group reported that they had pain affecting more than one lower limb site. The knee was the most commonly reported site for both traumatic and non-traumatic lower limb pain. The thigh was a commonly reported site for non-traumatic pain, while the ankle-foot was a common site for traumatic pain (Table 1). Traumatic injuries were reported by 34% (N = 54) of children with regional lower limb pain, and by 32% (N = 52) of children with combined lower limb pain.

### Pain interfering with daily activities

Out of 158 children who had regional lower limb pain, 70% (N = 110) reported at least one disability attributed their pain, 40% (N = 63) had pain that disturbs walking > 1 km, 30% (N = 47) had pain that disturbs their sleep. In addition, 27% (N = 42) reported that their pain interferes with their physical exercise classes, 18% (N = 28) had their pain interfering with hobbies and 4% (N = 7) reported that their pain interferes with their sitting comfort. School absence due to pain, for one or more days during the preceding 3 months, was reported by 19% (N = 30) of these children who reported frequent pains in their lower extremity.

### Factors associated with lower limb pain

In the univariate analysis, children aged 9–10 years had approximately 25% higher risk of lower limb pain compared with those aged 11–13. All psychosomatic symptoms (headache, abdominal pain, and depressive feelings, difficulty falling asleep, day tiredness and waking up during nights) were positively associated with occurrence of lower limb pain. Children who exercised vigorously (5–7 times a week) had approximately twice the risk of lower limb pain compared to those who exercised less than 3 times a week. In the multivariate analysis, age, headache, abdominal pain, depressive feelings, day tiredness, and vigorous exercise remained statistically significant (Table 2).

### Subjective disabilities and school absence related to traumatic and non-traumatic lower limb pain

Children with regional traumatic lower limb pain had a significantly higher subjective disability index than children with regional non-traumatic lower limb pain (t = 2.36, P = 0.02). Traumatic pain interfered with daily activities more than non-traumatic pain, with the exception of sleeping. There was no statistically significant difference in school absence between the children with traumatic and non-traumatic pain (Table 3).

### Risk factors for traumatic and non-traumatic lower limb pain

All psychosomatic factors were positively associated with lower limb pain in both groups. However, in the multivariate analysis, day tiredness was the only psychosomatic symptom associated with traumatic pain, while headache, depressive feelings and day tiredness were positively related to non-traumatic pain (Table 4). In the univariate analysis, there was a significant gender difference in traumatic pain experience, with males reporting more injury-induced pain in their lower extremity than girls. No relationship was found between physical activity/physical fitness and occurrence of non-traumatic pain in the lower extremity. On the other hand, both Vigorous exercise and high physical fitness were significantly and independently associated with traumatic lower limb pain. Furthermore, a significant synergistic interaction was found between these two factors (p = 0.015). The predictive effect of high physical fitness on occurrence of traumatic lower limb pain was stronger as the amount of exercise performed increased. The odds ratios of high physical fitness on occurrence of traumatic pain increased steadily from 2.13 (95% CI 1.12–4.42) in children who practiced infrequent exercise, to 3.35 (95% CI 1.22–5.88) in children who practiced moderate exercise, to 7.54 (95% CI 0.86–10.72) in children who practiced vigorous exercise. Similar to the crude results, the age of the preadolescents was inversely associated with both traumatic and non-traumatic lower limb pain. Hypermobility was not associated with both traumatic and non-traumatic pain and no statistically significant interaction were found between physical exercise and hypermobility on occurrence of both conditions. However, children with regional knee hypermobility were less likely to have traumatic pain compared to children with normal joint laxity (OR = 0.59 [95% CI 1.22–5.88]).

## Discussion

In our study, the knee was the most common site of pain in the lower limb, followed by the ankle and foot. This is in accordance with previous studies in children and adolescents [24,25]. The knee was also the most common location of pain due to a direct trauma, as has been reported previously [26]. About half the children who had lower limb pain reported pain also in other musculoskeletal location. This figure is substantially higher than the 19% prevalence of musculoskeletal pain in those without lower limb pain. This finding is consistent with previous surveys which have demonstrated frequent co-occurrence of different musculoskeletal complaints [12,27].

On the basis of our cross-sectional study, lower limb pain – both traumatic and non-traumatic- was more common among the younger age group (less than 11 years old) than among the older age group (from 11 to 13 years old). This contradicts the previous study by Vähäsarja, showing a clear increase in prevalence of knee pain from 4% in the 9–10 olds to 19% in the 14–15 olds schoolchildren [7]. However, this might be due to the different age distribution of our study population; which is composed of two age groups (9–10 and 11–12 years); and the wide case definition of lower limb pain used in our study (including pain in all lower limb area). More frequent non-traumatic lower limb pain among younger children (aged 9–10 years) is in accordance with the literature suggesting that "growing pains" mainly affects children aged 4 to 10 with occasional relapses in teenage years [8]. In this study, traumatic lower limb pain was more common among boys. Likewise, Smedbraten and co-workers found knee to be the only site in which pain was more common among boys [24].

Difficulty in walking long distances was the most common disability, reported by approximately 40% of children with lower limb pain. This is much higher than the 11% reported in a previous study about knee pain in schoolchildren [7]. However, the minimum distance was not specified in the later study. Disturbances of sleeping was the only disability associated to some extent more closely with non-traumatic than traumatic pain. This suggests that "growing pains" constituted a substantial proportion of non-traumatic pain.

Practicing vigorous exercise was associated with occurrence of non-specific lower limb pain. As mentioned earlier, conflicting results have also been reported about the role of physical exercise in occurrence of lower limb pain in schoolchildren [6,7,11]. we hypothesized that psychosomatic symptoms are associated only with non-traumatic lower limb pain, and that physical exercise is only related to traumatic lower limb pain, and this might be the reason for this conflicting results. As we expected, our stratified analysis showed that practicing vigorous exercise was associated with pain in the traumatic group but not in the non-traumatic group. After adjusting for physical activity and all other baseline predictors in our multivariate analysis, day tiredness was the only significant psychosomatic factor in the traumatic group. Day tiredness might have been a consequence of pain experience rather than had psychological origin. We have used the term "psychosomatic" to refer to symptoms that are chiefly considered to be expressions of psychological stress rather than manifestations of organic disorders based on previous research in children [28,29]. The same term was used in our previous reports [19,30] as well as by other researchers [31,32]. However it might be argued that some of these symptoms were manifestations of lower limb pain rather than related to emotional problems in childhood.

High level of physical fitness was independently associated with the occurrence of traumatic lower limb pain and practicing vigorous exercise was a significant effect modifier for this relationship. These results seem to contradict previous findings by Knapik et al. who reported that musculoskeletal injuries in young soldiers were associated with lower aerobic fitness [13]. However, several factors may prevent a valid comparison between our results and those of the latter study. Firstly, the age groups and the study settings in these studies were different; secondly, Knapik et al. were studying the relationship between physical fitness and all kinds of musculoskeletal injuries, including overuse injuries. Overtraining injury is not caused by a direct trauma but is related to repetitive loading on musculoskeletal structures without sufficient recovery. It must be noted that in the current study we have not collected information about overuse injuries, as we assumed that children would find it hard to understand the exact meaning of such type of injury. We have categorized our children into two groups according to whether or not they have directly traumatized their lower limb area with pain. Children who might have had overuse injuries were not included in the traumatic group, but rather in the non-traumatic group, as they have not indicated a direct trauma to the lower limb in the pain questionnaire. It might be possible that the predictive roles of physical fitness on the development of overuse and traumatic musculoskeletal injuries are different. However, this possible explanation cannot be examined with our data, and hence further research is needed to find out if such a difference really exists.

Occurrence of traumatic and non-traumatic lower limb pain did not differ between children with and without hypermobility. This is similar to our previous finding [19], regarding occurrence of musculoskeletal pain in general. However, children with regional knee hypermobility reported significantly less traumatic pain than children with normal joint laxity. This inverse association was not explained by differences in physical exercise frequency between these two groups. These results go well with the previous observations that hypermobility is not associated with increased athletic injuries [33] and that stretching plays an important role in decreasing the rate of injury [34]. These studies as well as ours, challenge the recommendation that children with hypermobility should avoid strenuous physical activities.

The most important strengths of this study are its population based setting and classifying lower limb pain according to location and association with injuries. In addition, pain interference with the children's daily activities was assessed. Yet, we did not have information about anatomic-structural or psychosocial factors, which might have been linked to occurrence of lower extremity pain in children.

## Conclusion

Our study indicates that risk factors and consequences of non-traumatic and traumatic lower limb pain are not similar. Non-traumatic pain is correlated with headache, recurrent abdominal pain and sleeping problems, while the traumatic pain is associated with practicing vigorous exercise and high level of physical fitness. This dissimilarity is consistent with our previous finding that traumatic lower extremity pain in preadolescents has a more favorable long-term natural course compared to non-traumatic pain [35]. Including or excluding traumatic injuries in the case definition of lower limb pain in research work investigating its determinants in children will significantly affect the results, and this might be the reason for the discrepancy of previous research conclusions. These two conditions need to be treated as different disorders in future studies.

## Declaration of interests

The author(s) declare that they have no competing interests.

## Authors' contributions

Dr. Ashraf El Metwally had primary responsibility for data analysis, and writing the manuscript. Dr. Jouko Salminen supervised the design of the study and contributed to the writing of the manuscript. Dr. Anssi Auvinen participated in the analytic framework for the study, and contributed to the writing of the manuscript. Hannu Kautiainen participated in the analytic framework for the study. Dr. Marja Mikkelsson developed the protocol and questionnaire, designed the study, supervised and participated in data collection, and contributed to the writing of the manuscript.

## Pre-publication history

The pre-publication history for this paper can be accessed here:



## Figures and Tables

**Table 1:** Prevalence of traumatic and non-traumatic lower limb, knee, ankle-foot, thigh, leg and hip pain occurring at least once a week pain (proportions out of 1756 preadolescent schoolchildren)

Prevalence, N (%)	Traumatic lower limb pain	Non-traumatic lower limb pain	Both lower limb pain groups
Lower limb pain	105 (6.0)	216 (12.3)	321 (18.3)
Ankle-foot pain	32 (1.8)	154 (8.8)	186 (10.6)
Knee pain	37 (2.1)	181 (10.3)	218 (12.4)
Thigh pain	15 (0.9)	166 (9.5)	181 (10.3)
Leg pain	30 (1.7)	89 (5.1)	119 (6.8)
Hip pain	13 (0.7)	47 (2.7)	60 (3.4)

**Table 2:** Odds ratios and confidence intervals of lower limb pain by age, sex, psychosomatic symptoms, physical fitness, frequency of exercise and hypermobility using univariate and multivariate logistic regression analyses. All figures were adjusted for occurrence of other musculoskeletal pain symptoms.

**Variables**	Univariate analysis (OR [95% CI])	Multivariate analysis^© ^(OR [95% CI])
Sex		
Boys	Reference	
Girls	0.83 (0.65–1.07)	
Age (years)		
9–10	Reference	Reference
11–13	0.74 (0.57–0.95)	0.66 (0.38 – 0.82)
°Psychosomatic symptoms		
Headache	1.93 (1.48–2.54)	1.61 (1.09–2.22)
Abdominal pain	2.34 (1.72–3.05)	1.57(1.03–2.12)
Depressive feelings	2.29 (1.72–3.05)	1.63 (1.09–2.32)
Difficulty falling asleep	1.73 (1.31–2.27)	
Day tiredness	2.79 (1.37–2.49)	2.54 (1.74–3.01)
Waking up during nights	1.84 (1.37–2.49)	
* Regional knee hypermobility	0.86 (0.64–1.16)	
Frequency of exercise		
0–2	Reference	
3–4	1.27 (0.95–1.70)	1.25(0.77–1.64)
5–7	1.87 (1.31–2.67)	1.85(1.20–2.87)
Beighton score for hypermobility		
< 6	Reference	
6–9	2.10 (0.86–5.05)	
^a^Vo_2 _max		
Average	Reference	
Low	0.79 (0.56–1.12)	
High	1.29 (0.93–1.77)	

^° ^Reported in a frequency of at least once a week.

^© ^Using backward stepwise procedure. Only entered variables shown. Results based on data from 1199 schoolchildren who completed the pain questionnaire and tested for both hypermobility and physical fitness.

* Not entered in the multivariate model.

^a ^Measured in ml/kg/min, categorized into 3 groups (below the 25^th ^percentile (low), between the 25^th ^and the 75^th ^percentiles (average), and above the 75^th ^percentile (high)

**Table 3:** Subjective disabilities and school absence in preadolescents with traumatic-and non-traumatic lower limb pain

*Variable*	Traumatic lower limb pain N = 54	Non-traumatic lower limb pain N = 104	P
*Subjective pain disabilities, N (%)*			
Pain disturbing sleeping	15 (27.7)	32 (30.8)	0.70
Pain disturbing sitting during class	3 (5.6)	4 (3.8)	0.62
Pain disturbing walking > 1 km	27 (50.0)	36 (34.6)	0.06
Pain disturbing physical exercise class	21 (38.9)	21 (20.2)	0.01
Pain disturbs hobbies	13 (24.1)	15 (14.4)	0.13
*Subjective disability index, Mean (SD)*	1.45 (1.1)	1.04 (1.0)	0.02
*School absence due to pain, N (%)*			
One day or more	12 (22)	18 (17)	0.52
1–2 days	9 (17)	11 (11)	0.31
3–7 days	2 (4)	5 (5)	1.0
> 7 days	1 (2)	2 (2)	1.0

**Table 4:** Odds ratios of traumatic and non-traumatic lower limb pain by sex, age, psychosomatic symptoms, hypermobility, frequency of exercise and physical fitness estimated by logistic regression analysis.

**Variables**	Univariate analysis [OR (95% CI)]		Multivariate analysis ^© ^[OR (95% CI)]	
	Traumatic	Non-traumatic	Traumatic	Non-traumatic
Sex (reference males)	0.62 (0.42–0.94)	0.98 (0.73–1.32)		
Age (years)				
9–10	Reference	Reference	Reference	Reference
11–13	0.75 (0.55–0.98)	0.69 (0.51–0.94)	0.64 (0.41–0.96)	0.67 (0.44–0.91)
^°^Psychosomatic symptoms				
Headache	1.56 (1.01–2.40)	2.14 (1.55–2.94)		1.88 (1.32–2.75)
Abdominal pain	2.40 (1.53–3.77)	2.28 (1.63–3.20)		
Depressive feelings	1.98 (1.26–3.12)	2.42 (1.75–3.37)		1.74 (1.19–2.76)
Difficulty falling asleep	2.18 (1.43–3.33)	1.50 (1.09–2.08)		
Day tiredness	2.83 (1.83–4.38)	2.76 (2.01–3.79)	2.81 (1.72–4.55)	1.98 (1.29–2.87)
Waking up during nights	2.14 (1.36–3.36)	1.73 (1.22–2.45)		
Beighton score for hypermobility				
Less 6	Reference	Reference		
6–9	0.96 (0.43–2.15)	1.05 (0.59–1.85)		
Regional knee hypermobility*	0.59 (0.35–0.99)	1.03 (0.73–1.44)		
Frequency of exercise				
0–2 times a week	Reference	Reference	Reference	
3–4 times a week	1.17 (0.72–1.88)	1.33 (0.95–1.86)	0.92 (0.64–1.66)	
5–7 times a week	2.62 (1.56–4.38)	1.48 (0.96–2.28)	2.04 (1.08–3.77)	
VO_2 _max				
Average	Reference	Reference	Reference	
Low	1.42 (0.88–1.91)	1.05 (0.70–1.58)	1.47 (0.89–2.24)	
High	2.85 (1.50–5.42)	0.96 (0.60–1.52)	12.97 (1.67–5.78)	

^© ^Using backward stepwise procedure. Only entered variables shown. Results based on data from 1199 schoolchildren who completed the pain questionnaire and tested for both hypermobility and physical fitness.

°Reported in a frequency of at least once a week.

* Not entered in the multivariate model

^a ^Measured in ml/kg/min, categorized into 3 groups (below the 25^th ^percentile (low), between the 25^th ^and the 75^th ^percentiles (average), and above the 75^th ^percentile (high)
